# Mitigating Moral Injury for Palliative Care Clinicians

**DOI:** 10.1089/pmr.2022.0062

**Published:** 2023-02-16

**Authors:** Anne G. Pereira, Mark Linzer, Leonard L. Berry

**Affiliations:** ^1^ Department of Medicine, Hennepin Healthcare, Minneapolis, Minnesota, USA.; ^2^ Department of Medicine, University of Minnesota Medical School, Minneapolis, Minnesota, USA.; ^3^ Mays Business School, Texas A&M University, College Station, Texas, USA.; ^4^ Institute for Healthcare Improvement, Cambridge, Massachusetts, USA.

**Keywords:** boundary spanning, moral distress, moral injury, palliative care

## Abstract

Palliative care clinicians (PCCs) in the United States face the combination of increasing burnout and a growing need for their services based on demographic changes and an increasing burden of serious illness. In addition to efforts to increase the number of PCCs and to train other clinicians in “primary palliative skills,” we must address the burnout in the field to address the growing gap between need for this care and capacity to provide it. To address burnout in PCCs, we must develop solutions with the unique contributors to burnout in this field in mind. PCCs are particularly susceptible to moral distress and moral injury faced by all clinicians, and these states are inextricably linked to burnout. We propose three solutions to address moral distress and moral injury in PCCs to reduce burnout. These solutions are grounded in the dilemmas particular to palliative care and in best evidence: first, to create space for PCCs to confront moral challenges head-on; second, to integrate ethics consultations into care of some patients cared for by PCCs; and third, to reassess care models for PCCs. These approaches can mitigate burnout and thus address the growing gap in our ability to provide high-quality palliative care for those patients in need.

## Introduction

A recent article in *Health Affairs* discusses the profound dilemma in sustaining the U.S. palliative care workforce.^1^ The authors argue that with 8000 palliative care clinicians (PCCs) in 2019 (an average of 160 PCCs per state), 40% age 55 years or older, and 33% being burned out, the specialty is facing an imminent “workforce valley.” At the same time, the need for PCCs has risen dramatically; in the authors' estimation, they may be needed to care for up to 12% of Medicare beneficiaries with multiple complex medical conditions. This workforce valley and growing need will combine to create unmanageable workloads for specialty PCCs.

PCCs provide relationship-based care for people facing serious illness, focusing on symptom management, anticipatory guidance in medical decision making, and interdisciplinary support for patients and their loved ones. To adequately populate the specialty, the challenges that lead to attrition among palliative care professionals must be assessed and addressed, so that the field can become more attractive and sustainable.

## The Present Burden

PCCs occupy a unique space in clinical medicine. They are “boundary spanners” between patients (and their families) and other clinicians. They commonly work in the tension-filled space between medicine as usual, which often seeks to prolong life at all costs, and the inevitability of decline and death in the context of serious medical illness. In oncology, for example, the oncologist is expert in treating tumors, whereas the PCC focuses on helping patients navigate illness amidst many, often dizzying treatment options. In addition to managing symptoms, PCCs identify patients' nuanced values, foster communication with other specialists,^2^ and develop a care plan aligned with patients' values (e.g., time at home, prolonging life, minimizing burdens on loved ones).

Although all clinicians have limited time for patients, PCCs have especially time-intensive duties as their workforce numbers dwindle and the referral base expands. At the most basic level, PCCs must learn what seriously ill patients and their families hold dear, helping them process complex choices about desired and undesired care.^3^ PCCs, therefore, are particularly susceptible to moral distress and the often-consequent more chronic *moral injury* that all clinicians face. Moral distress occurs when clinicians are unable to prevent medical actions they deem to be ethically inappropriate.^4^ Moral injury is an erosion of clinicians' trust from witnessing an egregious violation or a series of distressing events.^5^ Moral distress and injury go hand-in-hand with burnout,^6^ which threatens clinician and patient well-being.^7,8^

Solutions to mitigating moral injury for PCCs must be tailored to their boundary-spanning role. Those solutions nevertheless have the potential to generalize to other clinicians (e.g., subspecialists, nurses) and, thereby, create a foundation for identifying—and then reducing—the components and consequences of moral injury in the broader health care workforce, which has been demoralized and depleted by >2 years of the pandemic.^9^

## Boundary-Spanning Dilemmas

As boundary spanners, PCCs are steeped in the clinician side *and* the patient/family side of care planning. They understand the duty of medical professionals, along with the risk–benefit calculations of treatment options; they also intensely learn what patients and families value. When at their best, PCCs are as expert in a patient's values as they are in clinical medicine.

When PCCs embrace their core duty as boundary spanners, they may feel conflicted about clinicians' treatment recommendations that misalign with patient preferences—or worry about failing their duty to the patient/family.^10^ For example, PCCs may at times find themselves “standing by” and witnessing patient suffering in the face of continued treatment when their work has shown them that the patient would prefer more comfort-focused care. PCCs are prepared to engage in difficult conversations about treatments' burdens and benefits, yet the system is not well structured to facilitate such conversations.

When PCCs are unable to act in a patient's best interest, that dilemma can be a source of deep moral injury. We believe that this moral injury for PCCs can be eased as they simultaneously improve their communication with other subspecialists, nurses, and families, so that care plans ultimately benefit everyone, especially the patient.

## Three Solutions

Given PCCs' serious risk for moral injury, as well as the opportunity and need to enhance communication and care plans, we propose three evidence-based solutions, grounded in palliative care's particular dilemmas:

***1. Create space for PCCs to confront moral challenges head-on.*** Situations such as overtreatment of patients with advanced disease^5,6^ are opportunities for PCCs to provide care that has clear moral impact. Knowledge about the burdens and benefits of treatments should be weighed alongside ethical principles such as autonomy, beneficence, nonmaleficence, and justice in patient-related decision making.^11^ We, therefore, recommend an advanced disease practice standard whereby, much as a surgeon would communicate with a primary care clinician before operating on a patient, subspecialists involved in caring for the seriously ill patient would speak with the PCC, preferably before recommending additional burdensome treatments to patients and families.

This “upstream” approach would allow PCCs to “talk together” with colleagues (a highly regarded approach in chronic care management^2^) about patient priorities and values—and to offer suggestions from the patient's perspective on how to align care recommendations.^12^ It would provide more opportunities for reaching consensus, preventing situations when therapeutic momentum might contradict patients' or families' wishes, and avoiding the distress that PCCs feel when they must merely “stand by.” And it would streamline communication, allowing time for in-depth dialogue about care plans and fewer instances of “circling back” after misunderstandings or disagreements.

In rural Norway,^2^ researchers found that “formal and informal opportunities for talking together are a matter of providing coordinated collaborative patient care, including patient safety” for PCCs, oncology nurses, and general practitioners. Our proposal for a new practice standard aims to make these conversations the norm, whether in person in hospital-based palliative care settings or virtually when care teams are not colocated.

***2. Integrate ethics consultations.*** Maffoni has demonstrated that an ethical climate can profoundly affect clinical care for late-stage chronically ill patients, and for their health care providers.^13,14^ Medical ethicists can mitigate moral injury in situations with moral ambiguity.^11^ Just as a bed alarm is a practical safety measure for patients at risk for falls, patients with complex clinical profiles and uncertain prognoses are at risk for having their preferences overlooked. In cases where moral challenges loom, an ethically driven care review involving PCCs, ethicists, and key medical team members, before meeting with patient and family, can benefit everyone involved.^13–15^

Specifically, ethicists frame moral dilemmas in explicit terms, identifying opportunities for PCCs and the whole medical team to speak on behalf of a patient's best interests, even when the patient cannot.^11,15^ Although ethicists can guide parties to reach consensus on care plans for morally complex problems, they are called in only a tiny minority of such circumstances. We believe that to mitigate moral injury for numerous care providers (PCCs, nurses,^16,17^ and others), ethicists could assume an enhanced role in navigating the growing number of challenging situations that have no clear direction.

***3. Reassess care models for PCCs***. Health systems should acknowledge PCCs' boundary-spanning role as having high risk for moral distress and injury.^13^ Organizations could place metrics for burnout and moral injury on their dashboards, allowing senior leaders to monitor them alongside common measures such as quality, productivity, and safety. Such metrics could be calculated by department, thereby highlighting not only PCCs in their boundary-spanning role but also making visible comparable metrics in other disciplines that face their own set of challenges.

For example, given that moral injury and burnout can be associated with excessive work burdens, health systems could ask PCCs and other affected clinicians to regularly complete a simple work life dashboard metric: “I am experiencing work overload [not at all, somewhat, moderately, to a great extent].”^6^

Reducing PCCs' documentation and productivity expectations—to honor the time they need for reflection and complex face-to-face discussions with ever-increasing numbers of patients, families, and subspecialists—could increase their effectiveness and morale.^3^ To reduce PCCs' computer-facing time and increase patient-facing time, we propose using templated notes—including narrative, video-recorded, or dictated-for-transcription summations—and billing by hours spent navigating care wishes with patients facing serious illness. Cross-training other clinicians to stand in for PCCs in busy times could leverage PCCs' unique skill sets and temporarily expand the palliative care workforce.

The COVID-19 pandemic has been a watershed moment for medicine to mitigate clinicians' moral injury and burnout. Given the imminent need for growth in palliative care,^1^ burnout-related attrition is a pressing concern, as is the ability to recruit new colleagues to the field if they see overworked, depleted palliative specialists. PCCs intensely assess the needs of patients whose lives may soon end, as well as needs of patients not imminently dying but whose quality of life will fundamentally change due to late-stage illnesses. As boundary-spanning clinicians, PCCs must have their susceptibility to moral injury addressed if we want their careers to be sustainable—and to make moral rewards for PCCs more fulfilling for themselves, their colleagues, and the patients and families they serve.

## Abbreviation Used

PCCs: palliative care clinicians
